# Supplementary material for: Predicting visual function by interpreting a neuronal wiring diagram
Source: Nature. 2024 Oct 2;634(8032):113–23. doi: 10.1038/s41586-024-07953-5 (PMC11446822; doi:10.1038/s41586-024-07953-5)
Supplement: Supplementary file 6 — CRF and ERF predictions for individual TmY4 and TmY9 cells. Analogous to Supplementary Data 3, but for TmY target types. Shown are the top four monosynaptic pathways, the strongest pathway passing through each of the top ten intermediary types (ranking from Extended Data Fig. 7), and the trisynaptic pathway Tm1–TmY–Dm3–TmY (see the section entitled Prediction of spatial normalization). [file 41586_2024_7953_MOESM6_ESM.zip › DataS4/TmY9qΓèÑ/720575940635760350.pdf]

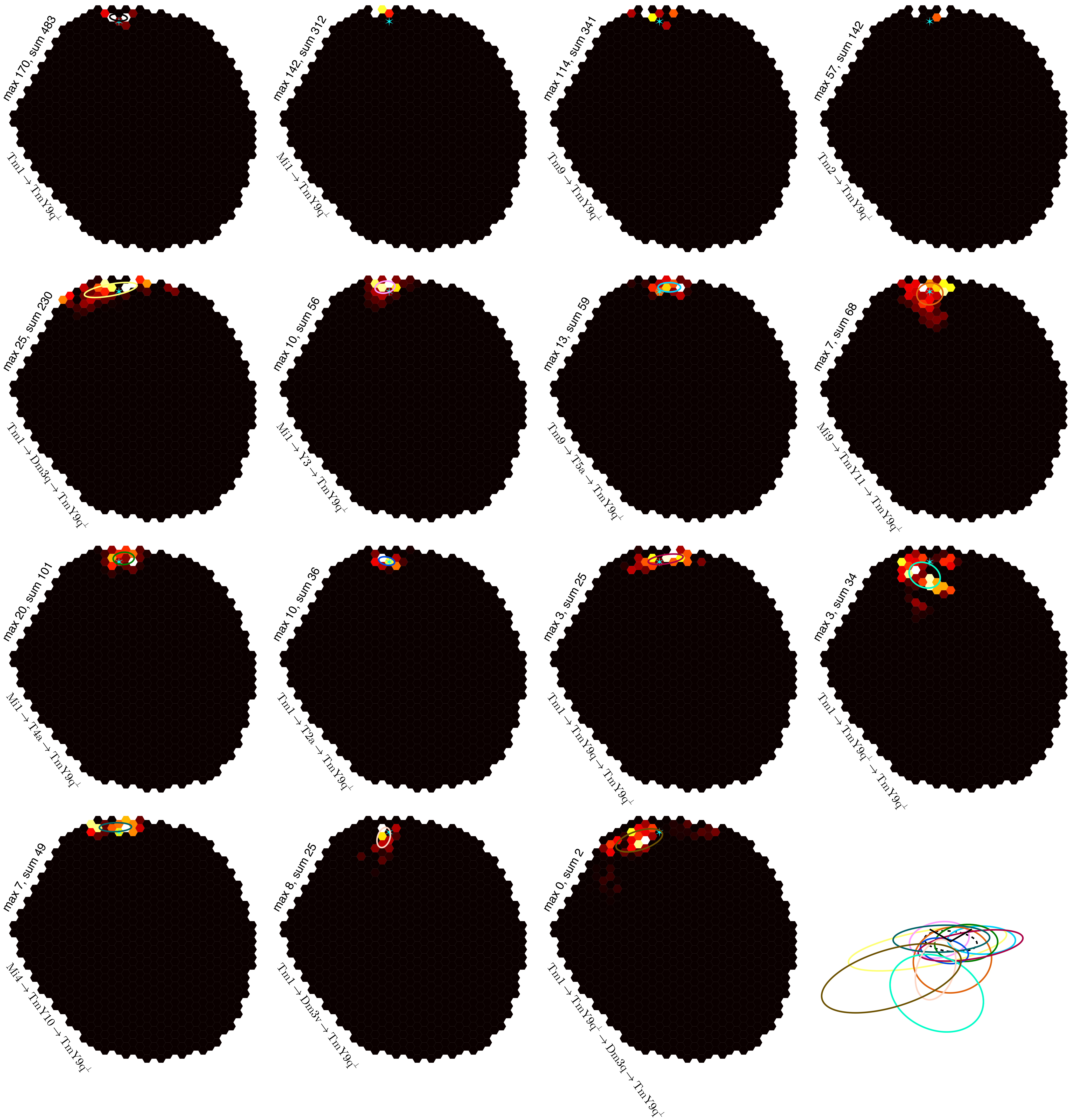
max 170, sum 483
Tm1 → TmY9q⊥
max 142, sum 312
Mi1 → TmY9q⊥
max 114, sum 341
Tm9 → TmY9q⊥
max 57, sum 142
Tm2 → TmY9q⊥
max 25, sum 230
Tm1 → Dm3q → TmY9q⊥
max 10, sum 56
Mi1 → Y3 → TmY9q⊥
max 13, sum 59
Tm9 → T5a → TmY9q⊥
max 7, sum 68
Mi9 → TmY11 → TmY9q⊥
max 20, sum 101
Mi1 → T4a → TmY9q⊥
max 10, sum 36
Tm1 → T2a → TmY9q⊥
max 3, sum 25
Tm1 → TmY9q → TmY9q⊥
max 3, sum 34
Tm1 → TmY9q⊥ → TmY9q⊥
max 7, sum 49
Mi4 → TmY10 → TmY9q⊥
max 8, sum 25
Tm1 → Dm3v → TmY9q⊥
max 0, sum 2
Tm1 → TmY9q⊥ → Dm3q → TmY9q⊥
